# Assessing the Accuracy and Feasibility of a Refractive Error Screening Program Conducted by School Teachers in Pre-Primary and Primary Schools in Thailand

**DOI:** 10.1371/journal.pone.0096684

**Published:** 2014-06-13

**Authors:** Kanlaya Teerawattananon, Chaw-Yin Myint, Kwanjai Wongkittirux, Yot Teerawattananon, Bunyong Chinkulkitnivat, Surapong Orprayoon, Suwat Kusakul, Supaporn Tengtrisorn, Watanee Jenchitr

**Affiliations:** 1 Health Intervention and Technology Assessment Program (HITAP), Nonthaburi, Thailand; 2 Department of Ophthalmology, Samut Prakan Hospital, Samut Prakan, Thailand; 3 Department of Pediatric Ophthalmology, Queen Sirikit National Institute of Child Health, Bangkok, Thailand; 4 Department of Ophthalmology, Surat Thani Hospital, Surat Thani, Thailand; 5 Department of Ophthalmology, Lamphun Hospital, Lamphun, Thailand; 6 Department of Ophthalmology, Nakhon Phanom Hospital, Nakhon Phanom, Thailand; 7 Department of Ophthalmology, Prince of Songkla University, Songkla, Thailand; 8 Faculty of Optometry, Rangsit University, Bangkok, Thailand; University of Sydney, Australia

## Abstract

**Introduction:**

As part of the development of a system for the screening of refractive error in Thai children, this study describes the accuracy and feasibility of establishing a program conducted by teachers.

**Objective:**

To assess the accuracy and feasibility of screening by teachers.

**Methods:**

A cross-sectional descriptive and analytical study was conducted in 17 schools in four provinces representing four geographic regions in Thailand. A two-staged cluster sampling was employed to compare the detection rate of refractive error among eligible students between trained teachers and health professionals. Serial focus group discussions were held for teachers and parents in order to understand their attitude towards refractive error screening at schools and the potential success factors and barriers.

**Results:**

The detection rate of refractive error screening by teachers among pre-primary school children is relatively low (21%) for mild visual impairment but higher for moderate visual impairment (44%). The detection rate for primary school children is high for both levels of visual impairment (52% for mild and 74% for moderate). The focus group discussions reveal that both teachers and parents would benefit from further education regarding refractive errors and that the vast majority of teachers are willing to conduct a school-based screening program.

**Conclusion:**

Refractive error screening by health professionals in pre-primary and primary school children is not currently implemented in Thailand due to resource limitations. However, evidence suggests that a refractive error screening program conducted in schools by teachers in the country is reasonable and feasible because the detection and treatment of refractive error in very young generations is important and the screening program can be implemented and conducted with relatively low costs.

## Introduction

Refractive error is a major cause of visual impairment and the second-most common cause of blindness in the world [1]. On World Sight Day in 2006, the World Health Organization (WHO) revealed that 153 million people aged older than five years were visually impaired due to uncorrected distance refractive error [2]. It was also estimated that the productivity lost from refractive error worldwide is over USD 269 billion [3]. In children, the prevalence of refractive errors varies widely across countries. For example, the prevalence of refractive errors was reported in primary school children in rural Tanzania at less than 1% [4], 8% in Kathmandu (Nepal) [5], 15% in Malaysia [6], 37% in Hong Kong [7], and more than 50% in Singapore [8]. In Thailand, the 4^th^ National Survey of Blindness in 2006–07 estimated that 15 million people were living with visual impairment due to uncorrected refractive error [9]. The prevalence of refractive error in primary school children (6–12 years old) in Bangkok was recently reported at approximately 13% [10].

A refractive error is correctable with spectacles, contact lenses or laser surgery; spectacles are the most available and least expensive method. However, the Refractive Error Survey in Children (RESC) – a cross-country survey about refractive error in children - indicated that the coverage of refractive corrections is no more than 50% in most regions of the world [11]. Severe visual impairment from uncorrected refractive error not only reduces the quality of life for an individual but may also impede education, delay personality development, and obstruct career opportunities [12]. These outcomes can also cause economic burden on the family and society as a whole. A study in India showed that one-fifth of refractive error-related blindness resulted from uncorrected high refractive error during childhood which is preventable if proper screening and provision of spectacles are available [13]. Although the diagnosis and treatment of refractive errors is simple, access to these procedures is still problematic due to many factors such as the lack of conclusive evidence regarding the effectiveness of the screening method, limited resources, and inadequate eye-care services in many countries.

A WHO study titled “Global Magnitude of visual impairment caused by uncorrected refractive errors” suggested that the screening of children for refractive errors should be conducted at the community level and integrated into school health programs where training and information programs should also be designed for teachers and school health-care workers [11]. However, there is no common agreement on what the best screening strategy is because different countries have different levels of health infrastructure development and methods to engage young children and their parents. Another previous study examined different strategies for school children that were conducted by teachers in Africa, Asia, America, and Europe and found that all of the screening strategies combined with the provision of spectacles were very cost-effective [14]. Thus, this study focuses on the development of a system for the screening of refractive error by teachers to formulate a national policy on screening and correcting refractive errors in Thai children.

## Methods

### i. Ethics Statement

Researchers sent a letter to all parents regarding the details of the study before any type of screening was performed. The written consents from parents were obtained only for children with positive screening results so they could undergo further eye examinations at hospitals. Written consents were not necessary for the screening performed by teachers and health professionals as visual acuity (VA) screening is a standard practice recommended by the WHO and many governments around the world, and is therefore not a harmful procedure. All signed consents from parents were reviewed by teachers and researchers and the consents will be kept for 5 years from the date of October 2011. The researchers received ethical clearance from the ethical committee of Medical Science in July 2011 before the study was conducted.

### ii. Study Design and Procedure

A cross-sectional descriptive and analytical study was conducted from October 2011 to January 2012. A two-staged cluster sampling was employed to select four representative provinces from four regions, resulting in the eventual selection of 17 schools. The schools were selected based on two main factors: i) school size (the number of students per school is similar to the third column in Tables S1.2 and S1.3 in File S1), and ii) the willingness of teachers to participate in the study. This was to ensure that the size of participants (screened students) was in proportion to the total population of eligible children in each province. It is important to note that the teachers' willingness to participate in the study was not a main factor because we did not find any school that refused to participate in this study. For the selection of participants, we included all of the students from the selected schools. A total of 5,885 students from pre-primary (4–6 years) and primary school grades (7–12 years) and 223 homeroom teachers participated in the study. A detailed breakdown of the sample size calculation is given in Section S1 in File S1.

In October and November of 2011, a number of ophthalmologists and ophthalmic nurses conducted a one-day training session for pre-primary school teachers in each of the provinces which focused on how to perform VA tests. During the training, the tools that were needed to conduct the VA tests (i.e. the VA screening manual, testing charts, eye occluders, and pinhole occluders) were provided. All teaching-conducted VA testing took place within a month of the teachers having received the training. A research team comprising ophthalmologists and ophthalmic nurses then tested the same pre-primary and primary school students in all of the selected schools between December 2011 and January 2012 using the same tools. The research team subsequently referred all children who had PVA worse than 20/40 in either eye and other eye disorders such as strabismus, latent strabismus, and congenital ptosis, to undergo further examination at the local provincial hospital. The research protocol is provided in Section S2 in File S1.

### iii. Ophthalmic Examination

#### a) Visual Acuity Testing

Participants were tested for ‘presenting visual acuity’ (PVA) - where participants who own spectacles were tested while wearing them. Testing was conducted on both eyes using the relevant eye chart - the ‘Lea symbols distance visual acuity chart’ for pre-primary school children (4–6 years old), the ‘E chart’ for the first years of primary school (7 years old), and the ‘Snellen chart’ for the remaining primary school children (8–12 years old). For each eye, the PVA was calculated according to the number of symbols or letters read correctly from 20/200 to 20/20. The PVA level was determined at the threshold where the child was able to read more than half of the given line. Children with a PVA level of less than 20/40 in either eye were referred to the local hospital. The guidelines that were used to measure the PVA for each group of children are given in Table S3.1 in File S1.

The PVA was classified according to the WHO ICD 10 classifications as follows: mild or no visual impairment: equal to or better than 20/70; moderate visual impairment: worse than 20/70 - equal to or better than 20/200; severe visual impairment to blindness: worse than 20/200.

#### b) Eye Examination

All of the children who had taken the tests were then examined at schools by the trained ophthalmologists using the same screening protocol. Any children who had normal VA but displayed symptoms of an eye disorder that required further diagnosis and treatment such as strabismus, latent strabismus, and congenital ptosis were subsequently referred to the local provincial hospital.

#### c) Diagnostic Procedure

All participants that were referred to the local provincial hospital underwent a thorough ophthalmic examination by both a general and pediatric ophthalmologist (with written informed consent from the parents). Auto refraction was performed and the ocular alignment, external eye, and anterior segments were examined in all of the referred children. Cycloplegia and dilatation were induced three times at intervals of 5 minutes by instillation of cyclopentolate 1% eye drops in children who had PVA worse than 20/40 in either eye. Auto refraction and manual refraction were then performed 30 minutes after the instillation of the last drop, and the posterior segment was examined after dilatation. A pediatric ophthalmologist made the final diagnosis and prescribed proper spectacle power for individuals who required it free of charge. Section S4 in File S1 contains the definitions that were used to diagnose eye disorders. Section S5 in File S1 describes the criteria used for spectacle prescription.

### iv. Statistical Analysis

Statistical analyses were carried out using PVA data from the participants' worst eye because empirical evidence has revealed that treating the worse eye in children has substantial benefits, especially in amblyopia. Thus, this screening program aims to detect all of the eyes with abnormal visual acuity. In the analysis of screening accuracy (i.e. sensitivity, specificity, and detection rate), only children who failed the VA test and were referred to hospitals were included in the analysis.

### v. Data Management

A sensitivity analysis was carried out on 5,303 students - 1,132 in pre-primary and 4,171 in primary - all of whom underwent a PVA test conducted by both teachers and professionals. We excluded data from students who were only tested by one group.

### vi. Focus Group Discussion

A set of 16 focus groups were convened among parents and teachers between September and October 2012 to understand more about the feasibility and limitations of establishing a school-based refractive error screening program. For teachers, we asked for their opinion regarding the feasibility and willingness to participate in the screening program as well as related factors if implemented. For parents, we focused on their general awareness of refractive error in children, particularly their own, and their attitude towards school-based screening and further treatment. In every province, the focus group discussions were carried out according to the geographic location of the schools (whether they were located in an urban or rural area). Within each area, separate focus groups were held for parents and teachers. In every section, the teachers involved had varying rates of sensitivity value (low, medium, and high) and came from both pre-primary and primary sectors. For the parent groups, we invited the parents of children both with and without refractive errors. All interviews were recorded on audiotape and transcribed verbatim. The first and the fourth authors read all the Thai transcripts to explore the respondents' experience with children with refractive error as well as attitudes and acceptance of the school-based screening.

## Results

### Overview of the research findings

Out of the 5,885 participating students, 5,703 children were screened by teachers, 5,461 by professionals, and 5,303 by both groups. The average age of pre-primary school students was 5 years (SD ±0.9) while that of primary school students was 9 years (SD ±1.8), with the male and female ratio being nearly equal. The average number of students screened by each pre-primary school teacher was 22 (SD ±10) while that of each primary school teacher was 26 (SD ±13). These general characteristic can be seen in Table 1. Of all the students screened by professionals (n = 5,461), 624 (11.4%) were referred to ophthalmologists at provincial hospitals as a result of exhibiting PVA levels less than 20/40 in either eye and/or abnormal results following an eye examination. However, only 470 (8.6%) children went for further examination at the provincial hospital because some of the children did not obtain consent from their parents or some did not show up at the hospital on the appointed day. Among the children who completed the examination (n = 470), 425 children were diagnosed with at least one eye disorder and 363 students were diagnosed with refractive error. Of the students with refractive error, 226 students received spectacles and 138 students were trained for near point convergence exercise. Ten students who were deemed likely to require surgical interventions were referred to specialist centers. Finally, refractive amblyopia was seen in 36 students, representing a delay diagnosis and correction of refractive error.

**Table 1:** Age, gender, and number of students screened by each teacher.

	Age
Pre-primary school students	5 (SD ±0.9)
Primary school students	9 (SD ±1.8)

	Gender (Male: Female)
Pre-primary school students	694 (52%): 641(48%)
Primary school students	2,308 (51%): 2,242 (49%)

	Number of students screened by each teacher
Pre-primary school level	22 (SD ±10)
Primary school level	26 (SD ±13)

### Sensitivity and specificity of teachers' screening

**Figure 1:**
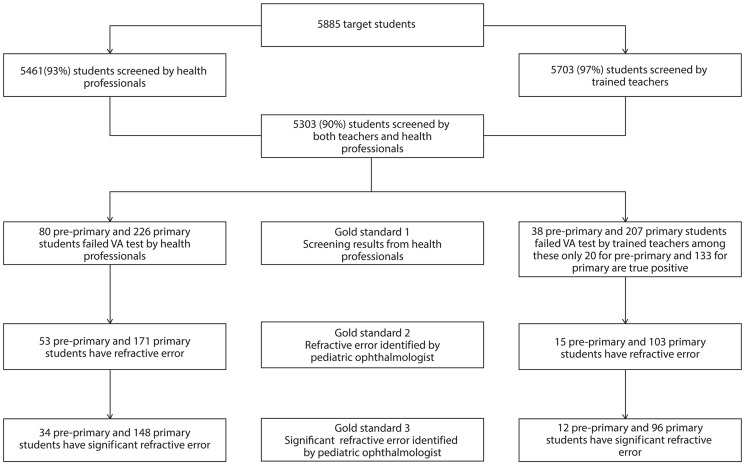
Selection of sample for sensitivity analysis.

Sensitivity values relating to the accuracy of pre-primary and primary school teachers' screening ability were assessed by comparing the accuracy of their diagnosis against three gold standards - Gold Standard 1, which refers to the accuracy rate of VA testing conducted by health professionals in a school setting; Gold Standard 2, which refers to the accuracy rate of VA testing conducted by a pediatric ophthalmologist at a local hospital following referrals resulting from testing in school; and Gold Standard 3, which refers to the accuracy rate of testing for significant refractive error (error requiring corrective eyeglasses) at a local hospital in addition to the Gold Standard 2 requirements. Figure 1 shows the selection process for the sensitivity analysis and outcomes of the screening process by both health professionals and teachers in relation to these three gold standards. Among the students who have low VA, 60 students out of 80 in pre-primary and 74 students out of 207 children in primary school students were misdiagnosed by the teachers as normal. They also incorrectly diagnosed 60 students out of 1,094 in pre-primary and 93 out of 3,964 in primary school students as low VA even though their VA was normal.

Sensitivity values among pre-primary school teachers (when measured against the three gold standards defined) were 25% (95% confidence interval of 23% to 27%), 28% (95% confidence interval of 26% to 30%), and 35% (95% confidence interval of 33% to 37%), while those of primary school teachers were 59% (95% confidence interval of 57% to 61%), 60% (95% confidence interval of 58% to 62%), and 65% (95% confidence interval of 63% to 67%). Specificity values were found to be high at around 97 to 98% in both groups. Table 2 describes the sensitivity and specificity values when compared with the three gold standards for both groups. In addition, results from a subgroup analysis found no significant difference across the categories of the examinee's age.

**Table 2:** Sensitivity and specificity value of the teachers with various levels of gold standards.

Gold standards	Sensitivity	Specificity
Participants	(95% confidence interval)	(95% confidence interval)
***Gold Standard 1: Screening results from the professionals***		
Pre-primary school teachers	25% (23% to 27%)	98% (97% to 99%)
Primary school teachers	59% (57% to 61%)	98%
***Gold Standard 2: Refractive error identified by pediatric ophthalmologist after screening by professionals***		
Pre-primary school teachers	28% (26% to 30%)	98% (97% to 99%)
Primary school teachers	60% (58% to 62%)	97%
***Gold Standard 3: Clinically significant refractive error identified by pediatric ophthalmologist after screening by professionals***		
Pre-primary school teachers	35% (33% to 37%)	98% (97% to 99%)
Primary school teachers	65% (63% to 67%)	97%

### Detection rate for teachers according to the severity of the visual impairment

**Figure 2:**
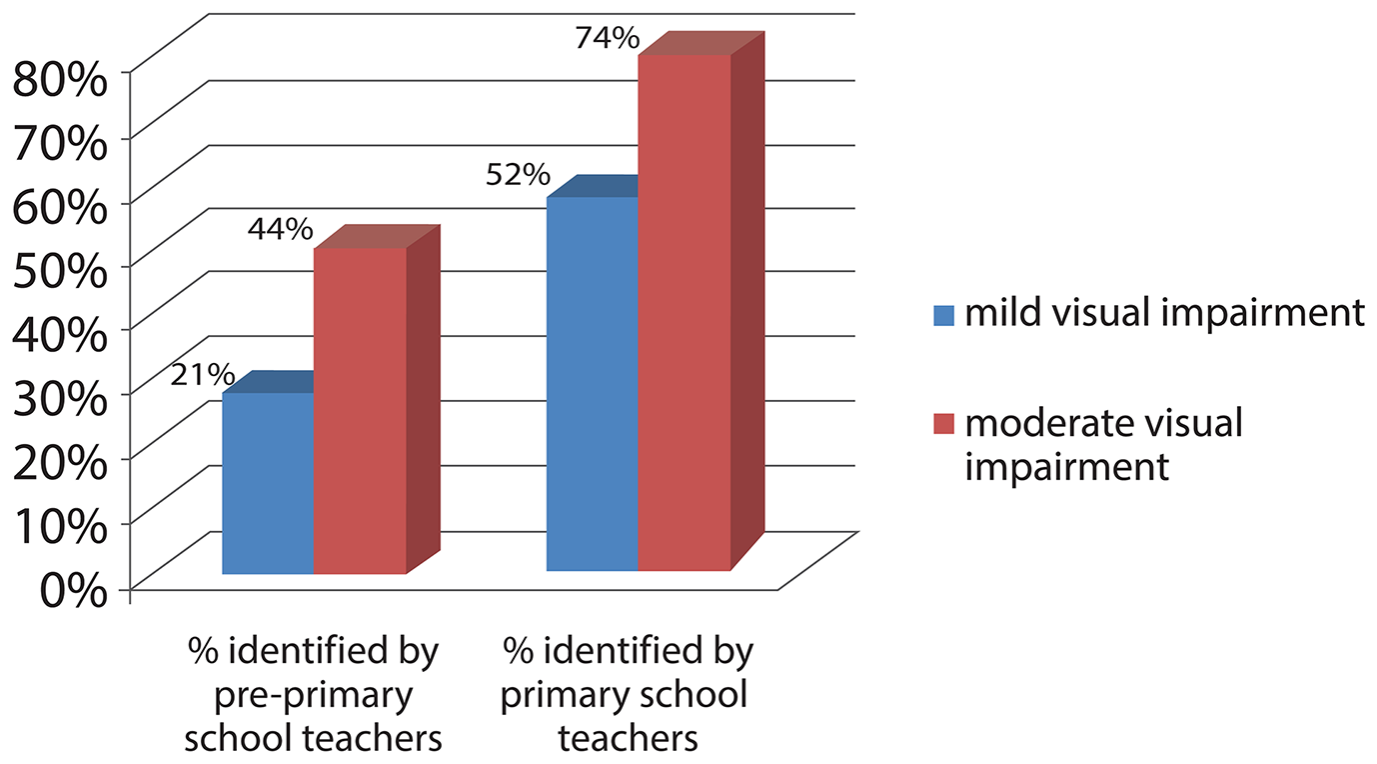
Detection rate of the teachers according to the severity of visual acuity level. Mild or no visual impairment: PVA equal to or better than 20/70; moderate visual impairment: PVA worse than 20/70 - equal to or better than 20/200; severe visual impairment to blindness: PVA worse than 20/200.

Among children with mild visual impairment, pre-primary school teachers were able to detect 8 cases out of 38 while primary school teachers detected 63 out of 122. Detection rates increased for children with moderate visual impairment - pre-primary school teachers were able to detect 8 cases out of 18 while primary school teachers could detect 40 out of 54. Although a number of children were diagnosed with severe visual impairment by the teachers, none of these children were found to have severe visual impairment upon professional examination. Figure 2 shows the detection rate by teachers for the different levels of impairment severity.

### The best cut-off point for defining visual impairment and referral for further investigation

The sensitivity of pre-primary school teachers abruptly rose to 74% when the cut-off point changed from 20/40 to 20/32. The estimated number of students receiving spectacles increased more than twofold with this cut-off point. Although the sensitivity of primary school teachers rose to 70% at the cut-off point of 20/30, the estimated number of students who were prescribed spectacles did not increase significantly. Tables 3 and 4 reveal the estimated number of pre-primary and primary students receiving spectacles at different cut-off points.

**Table 3:** Defining the best cut-off point for pre-primary school teachers' screening and estimated cases for a nationwide program.

Possible cut-off points	Sensitivity	Specificity	Estimated number of children referred for diagnosis*	Estimated number of children receiving spectacles*
20/20	93%	22%	1,264,085	46,401
20/25	76%	36%	1,026,454	42,183
20/32	74%	46%	887,250	42,183
20/40	25%	98%	53,432	16,873
20/50	16%	99%	30,934	12,655
20/64	6%	100%	11,249	7,031
20/80	1%	100%	5,624	1,406
20/100	1%	100%	1,406	1,406
20/126	0%	100%	0	0
20/160	0%	100%	0	0
20/200	0%	100%	0	0

* Hypothetical situation for 1,591,704 pre-primary school students [*28*] *; children receiving spectacles are the children who have significant refractive error.*

**Table 4:** Defining the best cut-off point for primary school teachers' screening and estimated cases for a nationwide program.

Possible cut-off points	Sensitivity	Specificity	Estimated number of students referred for diagnosis*	Estimated number of students receiving spectacles*
20/20	81%	79%	1,168,923	147,848
20/30	70%	92%	549,810	133,987
20/40	59%	98%	239,098	110,886
20/50	37%	99%	145,538	77,389
20/70	13%	99%	61,218	27,721
20/100	3%	99%	32,342	8,085
20/200	0%	100%	3,465	0

*Hypothetical situation for 4,817,764 students [*28*] *; children receiving spectacles are the children who have significant refractive error.*

### Focus group discussion among the teachers

The data collected as part of the focus groups indicated that teachers who screened their homeroom students did not feel that conducting the examinations yearly was a burden; instead, many felt proud to be able to help their students. However, some teachers lacked confidence in their ability to screen because of their perceived lack of experience. They requested that a significant period of time be given to the VA measurement training workshop - which was found to be the most important part of screening program - so that they had sufficient time to practice the screening techniques. As such, it is recommended that the training workshop should be provided at least once a year. Although the VA screening manual is very useful, it cannot be used alone without training. Pre-primary school teachers found that screening in very young children was very complicated, took a longer time, and required more patience. As such, it is recommended that at least two people (one teacher and one assistant) conduct the screening among children in this age group. Teachers often repeated measurements when students indicated a visual impairment to be sure that they were assessing the students appropriately. When teachers encountered problems with examining the children, they tended to ask another teacher to help them. Payment was not found to be an important incentive but teachers did indicate that the provision of an extra payment might encourage rapid and willing screening, although it was not a prerequisite for their willingness to conduct the screening.

Teachers found that children who had refractive error and always wore spectacles had better behavior when studying or playing at school than those who needed to wear spectacles but did not. Reasons given for not wearing spectacles included the risk of being teased by friends, the practical annoyance of wearing spectacles, unawareness among parents of the child's need or an unwillingness on the part of parents for their child to wear spectacles, the feeling that spectacles did not fit onto the child's face, or the fact that the spectacles had been lost. Most teachers believed that parents were an important part of the screening program. As such, it is vital that parents should be made aware of the risks and symptoms of refractive error in their children. This evidence suggests that an in-school teacher-led screening program will be successful and useful if it is built on a foundation of multidisciplinary cooperation between all stakeholders including policy-makers, local authorities, local hospitals, ophthalmologists, nurses, teachers, and parents.

### Focus group discussion among the parents

Most parents whose children were found to have a visual impairment had never suspected that their child might be experiencing difficulties with their sight, even when they observed certain behaviors such as watching TV or reading books very close up or when their child's writing was well outside the lines. Most parents never considered that these behaviors might be related to refractive error; instead, most saw them as quirks of childhood that would disappear as their child grew up. In addition, the majority of parents were under the impression that refractive errors were health problems that only happened to adults and the elderly; indeed, a few thought that spectacle-wearing made the child's visual problem worse and made the child look unintelligent.

Only very few parents had previously brought their children to a local optician or ophthalmology clinics/hospitals upon recognizing that their children might have visual problems. Despite the fact that spectacle costs are lower at ophthalmology clinics or hospitals than at local opticians, many parents preferred to bring children to a local optician rather than an ophthalmology clinic or a hospital because local opticians are regarded as more convenient, e.g. they are usually located nearer to their home, there is relatively little waiting time required, and - as a result of significant TV advertising by many optician companies - the service is regarded as better by many parents.

Having been informed about this study, almost all parents expressed willingness to have their children participate in a school-based screening program. Furthermore, they also asked that teachers provide more information to them about the screening program so that they could cooperate further. Lastly, all parents were willing to pay for spectacles if it was found that their children needed them to correct refractive error, although the amount they were willing to pay per year varied from 500 to 3,500 Baht with an average of 1,000 Baht.

## Discussion

This study found that the prevalence of refractive error among Thai school children is 6.6%, similar to some Asian countries but lower than in Singapore and China [15]–[19]. While other countries are struggling in establishing a population-based refractive error screening for children [20]–[22], this study demonstrates that refractive error screening by teachers is accurate and feasible in Thailand. However, we suggest that the cut-off points used for teacher-conducted screening should be different from those used by health professionals - especially among pre-primary school teachers - to maximize effective diagnosis. In our study, although 58 students already used spectacles (equivalent to 26% of those who needed spectacles), only 14 of them (equivalent to 6% of the children who needed spectacles) had accurate spectacles. Without our school-based screening, 168 students with refractive error (including 36 students with refractive amblyopia) would have never been diagnosed. Given the fact that these 168 students were found to have clinically significant refractive error, it is almost certain that this would have adversely affected their ability to access opportunities for childhood development.

Our study reveals a significant willingness on the part of the teachers to perform the screening. In addition, parents expressed interest in having their children screened by teachers because they trust them and also understand that it is not possible for health professionals to screen every child given their limited numbers. As a result, we strongly believe that with proper training, teachers will be able to conduct an effective school-based refractive error screening program for pre-primary and primary school students, thereby offering significant potential benefits for childhood development. Thus, we believe that a program of this type should be promoted in many resource-limited settings.

Data from this study should also be examined in light of similar studies conducted in other countries on primary school screening. In Iran, for instance, the sensitivity and specificity of teachers' screenings are 37.5% and 92% (at the 20/25 cut-off); in China, the rates are 93.5% and 91.2% (at the 6/12 or 20/40 cut-offs); and in Tanzania, the rates are 80% and 91% (at the 6/12 or 20/40 cut-offs) [4], [23], [24]. No other study, however, has examined teacher-conducted screening in pre-primary school children; this is the first study evaluating the feasibility and accuracy of non-health professionals screening for refractive errors in this population group. Although the number of children with refractive errors screened by teachers was lower than that of health professionals screened, most of these missed cases were children with mild visual impairment, and therefore does not constitute a serious public health concern. Furthermore, the screening program should be performed annually in order to reduce the undetected cases from previous screenings as well as to find new cases.

In fact, due to inadequate resources, refractive error screening by health professionals in pre-primary and primary school aged children is not currently implemented in Thailand. Although this study shows that the detection rate of screening by pre-primary school teachers is relatively low compared to that of primary school teachers, the recommendation for refractive error screening for both pre-primary and primary school aged children is warranted given the importance of detection and treatment of refractive error in very young generations and the relatively low cost of the screening program. Moreover, Figure 2 indicates that the detection rate for moderate visual impairment among pre-primary school children is as high as 44%, though the detection rate for mild visual impairment is quite low at 21%. In addition, Tables 3 and 4 reveal that using a higher cut-off point (e.g. 20/30 instead of 20/40) can increase sensitivity and thereby reduce the number of missed children with refractive error at the expense of a considerable increase in the number of students referred, whereas the number of students receiving spectacles will not significantly rise particularly in primary school. As a result, readers who wish to apply this protocol for screening refractive error among children need to carefully consider a cut-off point appropriate to their situation.

Since Thailand has a very high school enrollment rate of 95% for pre-primary [25] and close to universal for primary school [26], the implementation of this school-based screening program is likely to be effective. It is estimated that 260,000 children who require spectacles would have access to them and a number of children with refractive amblyopia would be avoided if this program is implemented nationwide. The results of this study was presented to high ranking decision-makers at the Ministry of Public Health and National Health Security Office (NHSO) in early 2013 and it was agreed that the program would be scaled up into a nationwide program within the next five years [27]. The teachers' screening is currently taking place at pre-primary and primary schools in ten provinces.

Furthermore, based on our experience, it is possible to improve the accuracy of teachers' screening by providing longer training sessions, especially hands-on practice (our training offered only 10 minutes per teacher). Moreover, it is necessary to have at least one assistant to a pre-primary teacher who performs refractive error screening using the Lea chart. The reason for this is that the Lea chart is a picture chart where the children are required to select similar model objects (to what they saw in the Lea chart) and show to the teacher at the same time the teacher needs to point out the Lea chart that is 3 meters far away from the children. We also suggest that further research should be performed in order to improve the techniques and accuracy of measuring VA among very young children.

However, this study does have some limitations. First, the provinces were selected to represent four regions in Thailand, although the selection of the 17 schools was randomly assigned among schools that matched our inclusion criteria, i.e. the number of students and the willingness to participate in the study. Second, because the screening conducted by teachers and health professionals were performed a month apart, 582 (10%) of the students missed a screening session by one of the groups. Third, although professionals recommended that 624 of the students who screened positive should go to the provincial hospital, only 470 (75%) students actually underwent further examination. Fourth, concerning the possible missed cases with hyperopia or astigmatism, we recommended teachers to observe students' reading behavior as indicated in the screening manual. If abnormal behavior such as reading at a very close distance, squinting, or head-tilting is found, the teachers can then refer those students to hospitals for a comprehensive eye examination including cycloplegic refraction. Lastly, this study focuses only on the accuracy and feasibility of refractive error screening by teachers. It does not evaluate the impact of correcting refractive error in children - which will require a longer timeline - nor does it evaluate the validity of recommending annual evaluations of refractive error in children.

## Supporting Information

File S1 Section S1: Sample Size Calculation. Section S2: Research Protocol. Section S3: Guidelines for measuring VA and the tools used. Section S4: Definitions used for diagnosis of eye disorders. Section S5: The criteria for prescribing spectacles.
(DOCX)
