# Supplementary material for: Assessing the Accuracy and Feasibility of a Refractive Error Screening Program Conducted by School Teachers in Pre-Primary and Primary Schools in Thailand
Source: PLoS One. 2014 Jun 13;9(6):e96684. doi: 10.1371/journal.pone.0096684 (PMC4057069; doi:10.1371/journal.pone.0096684)
Supplement: File S1 — Section S1: Sample Size Calculation. Section S2: Research Protocol. Section S3: Guidelines for measuring VA and the tools used. Section S4: Definitions used for diagnosis of eye disorders. Section S5: The criteria for prescribing spectacles. (DOCX) [file pone.0096684.s001.docx]

**Supporting Information**

**S1: Sample Size Calculation**

**S2: Research Protocol**

**S3: Guidelines for measuring VA and the tools used**

**S4: Definitions used for diagnosis of eye disorders**

**S5: The criteria for prescribing spectacles**

S1

SAMPLE SIZE CALCULATION

Sample size in this project was calculated using following formula:

Z (α/2)^2^ x *p* (1 – *p*)

n =

*d*^2^

1.96^2^ x 0.058 x 0.942

n =

0.0116^2^

n = 1,560

Where

n = required sample size

α = probability of type I error
Z = confidence coefficient = (1 - α)

*p* = estimated prevalence of refractive error in primary school children
*d* = precision of estimation at 20% of *p*

The estimated prevalence of refractive error in primary school students is 0.058 according to the summary of national survey in Thailand. Using the formula above, we calculated the total sample size of 1,560 students. Cluster sampling was used to get the required number of sampling schools in each province. In this project, we defined the design effect as equal to 2 so the total sample size in this project for cluster sampling is:

(n_clus_) = 1,560 x 2

= 3,120 students

The total sample size was divided into 2 groups; a group of pre-primary students and a group of primary students. The number of students in each group was calculated based on the proportion of pre-primary students and primary students in the four provinces as shown in Table S1.1.

Table S1.1: Number of pre-primary students and primary students in the four provinces

| Province | Number of pre-primary students | Number of primary students | Total |
| --- | --- | --- | --- |
| Nakhon Phanom | 16,379 | 53,648 | 70,027 |
| Lamphun | 6,729 | 16,223 | 22,952 |
| Samut Prakan | 22,323 | 77,594 | 99,917 |
| Surat Thani | 15,429 | 57,121 | 72,550 |
| Total | 60,860 | 204,586 | 265,446 |

*Source: Office of the Basic Education Commission*

The sample size of pre-primary school children:

60,860

= X 3,120

265,446

= 715.34 students, estimated 715 students

The sample size of primary school children:

204,586

= X 3,120

265,446

= 2,404.66 students, estimated 2,405 students

The sample size in each province was calculated using the proportion of students in the four provinces. In creating the sampling school, the calculated sample size was divided by the mean of students per school in the four provinces. The number of sampling schools was shown in Tables S1.2 and S1.3.

Table S1.2: Sample size of pre-primary school children in the four provinces

| Province | Sample size | Number of students/number of schools * | Required sampling school |
| --- | --- | --- | --- |
| Nakhon Phanom | 192 | 37 | 5 |
| Lamphun | 79 | 44 | 2 |
| Samut Prakan | 263 | 110 | 2 |
| Surat Thani | 181 | 36 | 4 |

**Source: Office of the Basic Education Commission*

Table S1.3: Sample size of primary school children in the four provinces

| Province | Sample size | Number of students/number of schools * | Required sampling school |
| --- | --- | --- | --- |
| Nakhon Phanom | 631 | 120 | 5 |
| Lamphun | 191 | 104 | 2 |
| Samut Prakan | 912 | 402 | 2 |
| Surat Thani | 671 | 142 | 5 |

**Source: Office of the Basic Education Commission*

S2

Research Protocol

Figure S2.1: Research Protocol

S3

Guidelines for measuring VA and the tools used

Table S3.1: Guidelines for measuring VA and the tools used

| **Tools** | **Target group** | **Testing distance** | **Given lines in each type of chart** | **Cut-off point for referral** |
| --- | --- | --- | --- | --- |
| **Lea Symbols distance visual acuity test** | Pre-primary school children (4-6 yrs) | 3 meters | 20/20, 20/25, 20/32, 20/40, 20/50, 20/64, 20/80, 20/100, 20/126, 20/160, 20/200 | < 20/40 |
| **‘E’ chart** | Grade 1  (7 yrs) | 6 meters | 20/20, 20/30, 20/40, 20/50, 20/70, 20/100, 20/200 | < 20/40 |
| **Snellen chart** | Grade 2-6  (8 – 12 yrs) | 6 meters | 20/15, 20/20, 20/30, 20/40, 20/50, 20/70, 20/100, 20/200 | < 20/40 |

S4

Definitions used for diagnosis of eye disorders

***Myopia***: the cycloplegic refractive power is equal to or more than -0.50 D;

***Hyperopia***: the cycloplegic refractive power is equal to or more than +0.50 D;

***Astigmatism***: the cycloplegic refractive power is equal to or more than 1.00 D**;**

***Anisometropia:*** The condition in which the two eyes have unequal refractive power. One eye may be myopic (nearsighted) and the other hyperopic (farsighted) or one eye may be markedly stronger than the other;

***Exotropia*** is a form of ocular misalignment where the eyes are deviated outward;

***Esotropia*** is a form of ocular misalignment in which one or both eyes turns inward;

***Heterophoria*** is a type of eye condition in which the directions that the eyes are pointing are not consistent with each other. It is classified into 2 types:

***Exophoria*** is a form of heterophoria in which there is a tendency of the eyes to deviate outward; and

***Esophoria*** is a form of heterophoria in which there is a tendency of the eyes to deviate inward.

S5

The criteria for prescribing spectacles which is similar to criteria of significant refractive error:

According to accommodative power by age group as indicated in the below —

Table S5.1: Guidelines for refractive correction in infants and young children.

| **Condition Refractive Errors (diopters)** | | | |
| --- | --- | --- | --- |
| **Age <1 year Age <2 years Age 2-3 years** | | | |
| **Isoametropia** (similar refractive error in both eyes) |  |  |  |
| Myopia | -5.00 or more | -4.00 or more | -3.00 or more |
| Hyperopia (no manifest deviation) | +6.00 or more | +5.00 or more | +4.50 or more |
| Hyperopia with esotropia | +2.50 or more | +2.00 or more | +1.50 or more |
| Astigmatism | 3.00 or more | 2.50 or more | 2.00 or more |
| **Anisometropia** (without strabismus)*  Myopia | -4.00 or more | -3.00 or more | -3.00 or more |
| Hyperopia | +2.50 or more | +2.00 or more | +1.50 or more |
| Astigmatism | 2.50 or more | 2.00 or more | 2.00 or more |

NOTE: These values were generated by consensus and are based solely on professional experience and clinical impressions because there are no scientifically rigorous published data for guidance. The exact values are unknown and may differ among age groups; they are presented as general guidelines that should be tailored to the individual child. **Specific guidelines for older children are not provided because refractive correction is determined by the severity of the refractive error, visual acuity, and visual symptoms.**

*Threshold for correction of anisometropia should be lower if the child has strabismus. The values represent the minimum difference in the magnitude of refractive error between eyes that would prompt refractive correction.

***Source: Pediatric Eye Evaluations PPP – 2012. AAO Pediatric Ophthalmology/Strabismus PPP Panel, Hoskins Center for Quality Eye Care:* [*www.aao.org*](http://www.aao.org)

2) Considering co-morbid eye disease/disorder such as heterophoria, heterotropia, or associated symptoms etc.
